# Effects of aerobic, resistance and concurrent exercise on pulse wave reflection and autonomic modulation in men with elevated blood pressure

**DOI:** 10.1038/s41598-020-80800-5

**Published:** 2021-01-12

**Authors:** Paulo Farinatti, Alex da Silva Itaborahy, Tainah de Paula, Walace David Monteiro, Mário F. Neves

**Affiliations:** 1 Graduate Program in Exercise and Sports Sciences, University of Rio de Janeiro State, Rio de Janeiro, Brazil; 2 Graduate Program in Physical Activity Sciences, Salgado de Oliveira University, Niteroi, Brazil; 3 Clinic of Hypertension and Associated Metabolic Diseases, University of Rio de Janeiro State, Rio de Janeiro, Brazil; 4 Laboratory of Clinical and Experimental Pathophysiology, University of Rio de Janeiro State, Rio de Janeiro, Brazil; 5 Departamento de Clínica Médica, Hospital Universitário Pedro Ernesto, Centro Biomédico, Universidade Do Estado Do Rio de Janeiro, Boulevard 28 de Setembro 77/329, Rio de Janeiro, RJ 20551-030 Brazil

**Keywords:** Circulation, Physiology, Cardiology

## Abstract

The acute effects of exercise modes on pulse wave reflection (PWR) and their relationship with autonomic control remain undefined, particularly in individuals with elevated blood pressure (BP). We compared PWR and autonomic modulation after acute aerobic (AE), resistance (RE), and concurrent exercise (CE) in 15 men with stage-1 hypertension (mean ± SE: 34.7 ± 2.5 years, 28.4 ± 0.6 kg/m^2^, 133 ± 1/82 ± 2 mmHg). Participants underwent AE, RE, and CE on different days in counterbalanced order. Applanation tonometry and heart rate variability assessments were performed before and 30-min postexercise. Aortic pressure decreased after AE (− 2.4 ± 0.7 mmHg; *P* = 0.01), RE (− 2.2 ± 0.6 mmHg; *P* = 0.03), and CE (− 3.1 ± 0.5 mmHg; *P* = 0.003). Augmentation index remained stable after RE, but lowered after AE (− 5.1 ± 1.7%; *P* = 0.03) and CE (− 7.6 ± 2.4% *P* = 0.002). Systolic BP reduction occurred after CE (− 5.3 ± 1.9 mmHg). RR-intervals and parasympathetic modulation lowered after all conditions (~ 30–40%; *P* < 0.05), while the sympathovagal balance increased after RE (1.2 ± 0.3–1.3 ± 0.3 n.u., *P* < 0.05). Changes in PWR correlated inversely with sympathetic and directly with vagal modulation in CE. In conclusion, AE, RE, and CE lowered central aortic pressure, but only AE and CE reduced PWR. Overall, those reductions related to decreased parasympathetic and increased sympathetic outflows. Autonomic fluctuations seemed to represent more a consequence than a cause of reduced PWR.

## Introduction

Central arteries stiffening has been reported to increase the chance of developing left ventricular hypertrophy and myocardial ischemia^1^. For this reason, it is an independent predictor of cardiovascular mortality and morbidity and, therefore, a prognostic indicator of long-term cardiovascular health^2,3^. Measurements of pulse wave reflection (PWR) are often used as indirect markers of arterial stiffness^4^, resulting from factors as left ventricular ejection, elastic properties of large arteries, and wave reflections that occur at bifurcations points spread over the arterial tree^5,6^. The estimation of PWR and left ventricular afterload by assessing augmentation index (AIx) has been suggested as a prognostic indicator of adverse cardiovascular events^7,8^.

It has been shown that exercise training may lower vascular resistance and arterial stiffness, contributing to attenuate left ventricular afterload during systole, increasing diastolic myocardial perfusion^5^ and preventing cardiovascular events^9,10^. Improving arterial function through exercise is therefore of clinical importance. Accumulated evidence acknowledges that time-course adaptations in PWR result from chronic exercise. Aerobic training seems to reduce PWR (i.e., AIx and reflection magnitude), while the effects of resistance training are inconclusive, with trials reporting increasing, decreasing or no change in PWR and arterial stiffness outcomes^4,10–15^. The effects of acute exercise have been suggested as potential determinants of overall hemodynamic chronic adaptations, including arterial stiffness^4,16,17^, which is probably attenuated by the exercise mode^4,12,18,19^. However, research on the after-effects of different exercise modes (i.e., aerobic vs. resistance exercise) on PWR and arterial stiffness are scarce and produced unconclusive findings^4,12^.

The few trials examining the effects of acute exercise on PWR mostly included young individuals with normal blood pressure (BP)^4^. Moreover, BP at rest was not treated as a moderator of acute changes in arterial stiffness. This is remarkable, since resting BP levels probably influence wave reflection and arterial stiffness. In healthy young individuals, a large part of wave reflections takes place during diastole. In contrast, in patients with elevated BP, the aortic pressure during systole may increase due to augmented reflected waves^5^. Consequently, the acute patterns of central aortic pressure waves tend to be different in individuals with normal and elevated BP. A better comprehension of PWR after exercise in individuals with elevated BP could help to elucidate the relationships between acute and chronic effects of physical training in this population.

Furthermore, individuals with elevated BP are more likely to exhibit autonomic dysfunction^20^. A greater sympathovagal balance is acknowledged to increase vasoconstriction^20^, with a potential impact on aortic pressure and PWR^4,21,22^. Just to illustrate, since the increased heart rate is one of the variables lowering aortic pressure^23^, a reduction in wave reflection during postexercise recovery could suffer, at least in part, the influence of autonomic control. The level of peripheral vasoconstriction induced by sympathetic stimulation has been also shown to increase the reflected wave intensity, which seems to persist after exercise^24,25^. Additionally, acute changes in arterial stiffness are assumed to result from alterations in BP during exercise^26^. It is well accepted that BP responses postexercise are modulated by arterial distensibility and the timing of PWR, as well as by autonomic fluctuations^9,10,25,27,28^. Despite this, relationships between acute changes in PWR and cardiac autonomic activity after different exercise modalities are yet to determine, particularly in individuals with elevated BP^4^.

In short, investigations addressing how PWR and autonomic outcomes respond to different modalities of acute exercise in individuals with elevated BP might contribute to a better understanding of the impact of exercise training on the cardiovascular health in this population. Therefore, this study aimed to investigate changes in BP, PWR and autonomic modulation after acute aerobic (AE), resistance (RE), concurrent exercise (CE) in men with stage-1 hypertension. We hypothesized that changes in PWR time-course would depend on exercise modalities. Besides, we expected that they would be related to fluctuations in autonomic control, particularly sympathetic outflow.

## Methods

### Study participants

Participants were physically inactive men aged 18- to 50 years with stage-1 hypertension, recruited from an outpatient clinic at the Pedro Ernesto University Hospital from the State University of Rio de Janeiro. Inclusion criteria were: (a) Systolic BP between 120 and 139 mmHg and/or diastolic BP between 80 and 89 mmHg. Exclusion criteria were: (a) Use of drugs influencing physical performance, hemodynamic or autonomic responses; (b) Smoking; (c) Participation in exercise or nutrition programs within 6 months before the experiment; (d) Diabetes, dyslipidemia, liver dysfunction, chronic kidney disease, or thyroid dysfunction; e) Cardiovascular, respiratory, or muscle-skeletal disorders precluding physical exercise. All participants gave written informed consent before enrolling in the study, which was approved by the institutional ethics committee (CAAE: 0251.0.228.000-11).

### Study design

The experiment included seven visits to the laboratory, interspersed with at least 48-h intervals. On the first visit, we checked the inclusion and exclusion criteria through clinical examination. On the second visit, eligible individuals underwent venous blood collection and at-office BP assessment. On the third visit, a 15-repetition maximum-load test^29^ for the bilateral knee extension was performed, which was repeated after 60-min to verify test–retest reliability. After 48 h to 7 days, participants underwent maximal cardiopulmonary exercise testing (CPET). On the remaining visits, AE, RE, and CE were performed in a counterbalanced alternate order.

**Figure 1:**
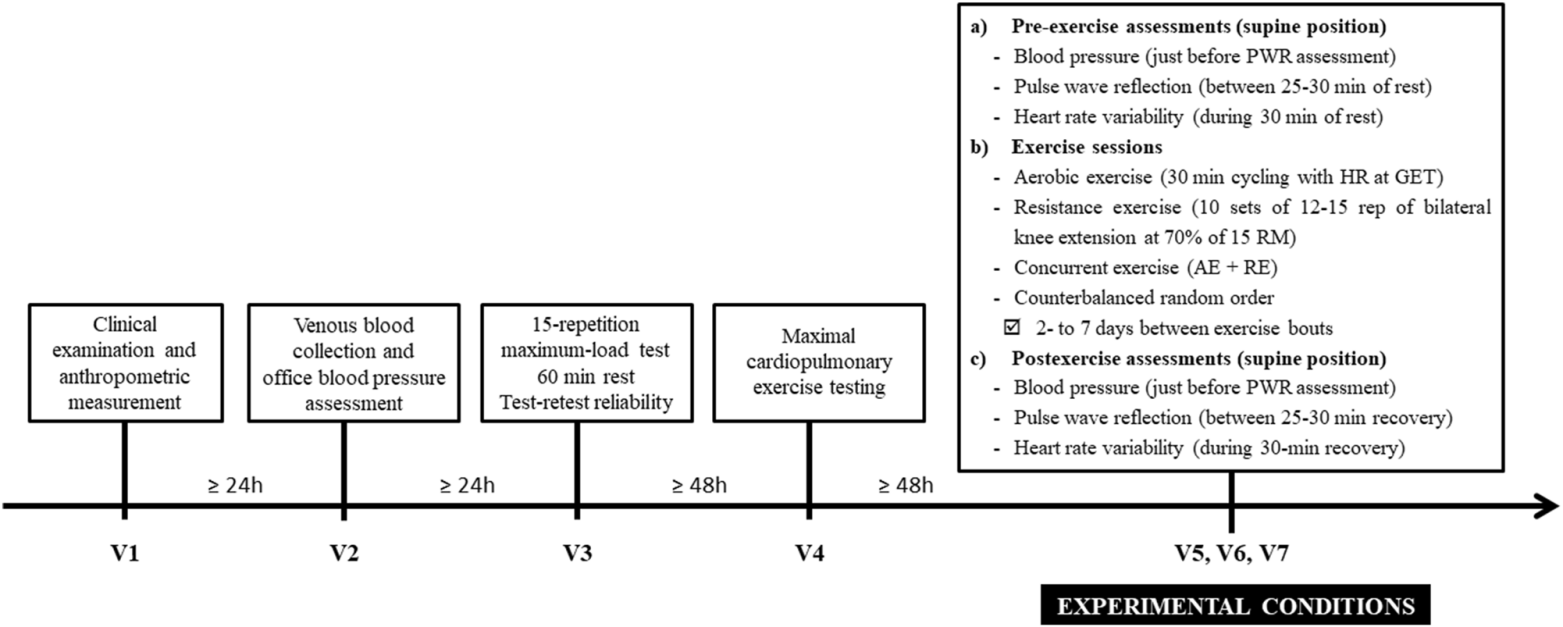
Study design.

All participants underwent the three exercise modalities, and experimental sessions were interspersed with washout intervals of 2- to 7 days. Hemodynamic and PWR outcomes were performed along 30 min pre and postexercise. Tests and interventions occurred between 8 and 12 a.m. to mitigate potential circadian effects. Prior to experimental sessions, participants were told to wear clothing and shoes consistent with sporting activities; to avoid alcoholic beverages or stimulants (coffee, chocolate, teas, etc.) up to 24 h before tests; and to avoid strenuous physical activities such as running, long walks, or weight training up to 48 h before testing. Figure 1 summarizes the study design.

### Biochemical analysis

A venous blood sample was collected after 12-h fasting. Total cholesterol, triglycerides, HDL-cholesterol, glucose, creatinine, and uric acid were analyzed using enzymatic methods, while LDL-cholesterol fraction was estimated by the Friedewald equation when triglycerides concentration did not exceed 400 mg/dL. Glomerular filtration rate (GFR) was estimated by the CKD-EPI equation^30^.

### Maximum cardiopulmonary exercise testing (CPET)

Maximal oxygen uptake (VO_2_max) was assessed through CPET performed on a cycle ergometer (Cateye EC-1600, Cateye, Tokyo, Japan), according to a ramp protocol designed to elicit maximal volitional effort. Initially, VO_2_max was estimated using a non-exercise model for healthy individuals aged 19–80 years^31^. The maximal load was individually calculated using the cycling equations from the American College of Sports Medicine^29^. Cycling cadence was maintained at 55 revolutions per minute. After a 3-min warm-up with no resistance, the ramp protocol began with 50% of the predetermined maximal load. The ramp has been programmed to last 10 min, ranging from 8- to 12-min.

Ventilatory exchanges were determined using a VO2000 analyzer (Medical Graphics, Saint Louis, MO, USA) with a silicone facemask (Hans Rudolph, Kansas, MO, USA). Gas exchange outcomes were 20-s stationary time-averaged, which provided a good compromise between removing data noise while maintaining the underlying trend. Immediately before testing, the metabolic cart was calibrated using a certified standard mixture of oxygen (17.01%) and carbon dioxide (5.00%), balanced with nitrogen (AGA, Rio de Janeiro, RJ, Brazil). The heart rate was measured continuously using a cardio tachometer RS800cx (Polar, Kempele, Finland) and beat-by-beat data were 20-s stationary time-averaged.

Tests were considered as maximal in the presence of at least three of the following criteria: (a) Maximum exhaustion defined by attaining the score 10 on Borg CR-10 scale; (b) 90% of predicted maximal heart rate [220 − age] or heart rate plateau (Δ heart rate between two consecutive work rates ≤ 4 beats/min); (c) VO_2_plateau (ΔVO_2_ between two consecutive work rates < 2.1 mL kg^−1^ min^−1^); (d) respiratory exchange ratio > 1.10^32^. Ambient temperature and humidity ranged from 21 to 23 °C and 55–70%, respectively.

### 15-repetition maximum test (15RM)

The load corresponding to 15RM was determined for the bilateral knee extension (Cybex VR2, Medway, MA, USA). Participants were instructed to mobilize the maximum load performing 15 repetitions of complete concentric/eccentric phases, according to procedures described elsewhere^33^. Test–retest reliability was confirmed after a 60-min interval by means of intraclass correlation (ICC = 0.93, *P* < 0.0001).

### Blood pressure and pulse wave reflection assessments

After 15 min of seated rest, BP was assessed five times with 3-min intervals between measurements^34^, using a semi-automatic device (Omron—HEM-433int, Bannockburn, IL, USA). We discarded the first value and recorded the average of the next four measurements. The same device measured casual BP before each exercise session, to check for possible fluctuations across different days. Aortic markers of PWR were derived from radial pulse wave analysis through applanation tonometry (SphygmoCor, AtCor Medical, Sydney, Australia). A tonometer (SPC-301, Millar Instruments, Houston, TX, USA) was used after calibration according to the previously measured brachial BP. The aortic waveform was processed from averaged recordings of 10 radial waveforms by a previously validated transfer function^35^. Applanation tonometry was applied within 25–30 min of pre and postexercise periods. Measurements were performed in duplicate, and average values were recorded.

We analyzed the following central markers of PWR: aortic systolic BP (aoSBP), aortic pulse pressure (aoPP), augmented pressure (AP), augmentation index (AIx), and augmentation index normalized to 75 bpm (AIx75). The AP is the increment in aoSBP above its first systolic shoulder. It reflects the contribution of wave reflection to systolic arterial pressure, corresponding to the reflected wave coming from the periphery to the center^36^. The maximal change in AP due to differences in systolic and diastolic pressures determines aoPP. The amplitude and timing of the reflected wave ultimately depend on the stiffness of vessels. Therefore, as a function of PWR, AP and aoPP largely depend on the pulse wave velocity, which in turn is dependent on arterial elasticity. Augmentation refers to the difference between the second and first systolic peaks of the central pressure waveform, and AIx is defined as augmentation expressed as a percentage of pulse pressure^12,37^. For this reason, it is acknowledged as a measure of systemic arterial stiffness derived from the ascending aortic pressure waveform^7,11,24^. On the other hand, heart rate also modulates AP. Longer ejection time and cardiac cycle under low heart rate conditions retard the reflected wave, thereby increasing AP and AIx. Hence, AIx and heart rate are inversely related^11^ and to avoid bias, AIx75 integrates a correction factor normalizing its value to heart rate^38^.

### Heart rate variability assessment

Cardiac autonomic modulation was evaluated through heart rate variability (HRV)^39^. The R-R intervals (iRR) were continuously registered by telemetry (Polar RS800cx, Polar Electro, Kempele, Finland). Data were downloaded with the Polar Precision Performance Software (Polar, Kempele, Finland), and averaged for each 5-min window (sampling frequency of 1000 Hz). Signal artifacts were filtered by excluding iRR values with differences of more than 30% of the preceding iRR. Before exercise, the participants remained in the supine position for 30 min and recordings of the last 5 min were retained for analysis (pre-exercise). HRV was also assessed during 30 min of postexercise recovery and analyzed at each 5-min. The first time point was at 10 min, to allow the participants to resume the supine position.

HRV analysis was performed in time and frequency domains using the Kubios HRV Analysis Software 2.0 (Biomedical Signal and Medical Imaging Analysis Group, Department of Applied Physics, University of Kuopio, Finland). The time-domain analysis consisted of measures of average RR intervals (iRR) and rMSSD (square root of the sum of successive differences between adjacent normal R-R intervals squared). In the frequency domain, the power spectrum density function was integrated into two frequency bands: (1) low-frequency power (LF: 0.04–0.15 Hz); and (2) high-frequency power (HF: 0.15–0.40 Hz)^39^. The HF was adopted as a marker of vagal modulation, whereas LF was considered as representative of the modulation of both sympathetic and parasympathetic nervous branches^39^. The spectral values were expressed as normalized units (n.u.)^40^.

### Experimental sessions

Exercise protocols complied with commonly prescribed bouts for cardiovascular health^29^. AE consisted of 30-min cycling with an intensity corresponding to heart rate at GET (± 2 bpm), preceded by 5-min warm-up (50–55 rpm and 30 W). RE included 10 sets of 12–15 repetitions of bilateral knee extension (leg press) with load corresponding to 70% of 15RM (2-min intervals between sets). The RE protocol included a single exercise because only this machine was available at the research facilities. The load and expressive number of sets and repetitions were defined based on results of previous studies, showing that in a similar exercise protocol involving large muscle mass, this combination was capable of acutely reducing BP and peripheral resistance^41,42^. Participants should entirely extend their knees in each repetition, to avoid the Valsalva maneuver, and not to use the hands as support to prevent the recruitment of forearm muscles (which could interfere with the applanation tonometry). In CE, participants performed 5-min warm-up on a cycle ergometer, followed by 10 sets of 12–15 repetitions of bilateral knee extension with the same load applied in RE, and 30 min of cycling at the same intensity applied in AE. A 5-min interval was allowed between RE and AE components. All sessions took place in a quiet temperature-controlled room (20–22 °C).

### Statistical analysis

Sample size was calculated a priori for α = 0.05 and β = 0.80, using the G*Power software 3.1.9.2^43^. A sample of 14 individuals was required for detecting significant differences with medium effect size (Cohen’s D = 0.30). Data normality was tested by the Shapiro–Wilk test and logarithmic transformation was applied whenever possible. Data are therefore presented as mean ± standard error. BP, PWR, and autonomic outcomes were compared between experimental conditions and pre vs*.* postexercise, employing a 2-way ANOVA for repeated measures, followed by Tukey post hoc verifications in the event of significant *F* ratios. Associations between PWR and HRV data were determined using Spearman correlations. All calculations were made using the Statistica 10.0 software (StatSoft, Tulsa, OK, USA), and statistical significance was set at *P* ≤ 0.05.

### Ethics approval

This study gained approval from the Ethics Committee Board from the Pedro Ernesto University Hospital at the University of Rio de Janeiro State (RJ, Brazil, CAAE: 0251.0.228.000-11). All procedures were carried out in line with the Declaration of Helsinki.

### Consent to participate

All participants provided signed informed consent before participation in the study.

## Results

Table 1 presents data of clinical characteristics, hemodynamic outcomes, and blood analysis, as well as peak oxygen consumption, and maximum heart rate during CPET.

**Table 1:** Characteristics of the sample (n = 15).

Characteristic	Mean (SE)
Age (years)	34.7 (2.5)
BMI (kg/m^2^)	28.4 (0.6)
Abdominal circumference (cm)	96.6 (1.7)
**Hemodynamic**	
SBP (mmHg)	133 (1)
DBP (mmHg)	82 (2)
MAP (mmHg)	99 (1)
HR (bpm)	66 (2)
PP (mmHg)	51 (2)
**Blood biochemistry**	
Hemoglobin (g/dL)	13.8 (0.3)
Glycemia (mg/dL)	82 (2)
Creatinine (mg/dL)	0.9 (1.7)
eGFR (mL/min/1.73 m^2^)	115 (4)
Uric acid (mg/dL)	6.2 (0.2)
Total cholesterol (mg/dl)	189 (10)
HDL-cholesterol (mg/dL)	43 (3)
LDL-cholesterol (mg/dL)	129 (9)
Triglycerides (mg/dL)	125 (19)
**CPET**	
VO_2 peak_ (ml/kg/min)	27 (1)
HR_peak_ (bmp)	170 (3)

Values expressed as mean (SE).

BMI, body mass index; SBP, systolic blood pressure (brachial); DBP, diastolic blood pressure (brachial); MAP, mean arterial pressure (brachial); HR, heart rate; PP, pulse pressure; CPET, cardiopulmonary exercise test; VO_2peak_, peak of oxygen uptake. HR_peak_, maximum heart rate; eGFR, estimated glomerular filtration rate; HDL, high-density lipoprotein, LDL, low-density lipoprotein; CPET, cardiopulmonary exercise test; VO_2 peak_, peak of oxygen consumption; HR_peak_, maximum heart rate.

**Figure 2:**
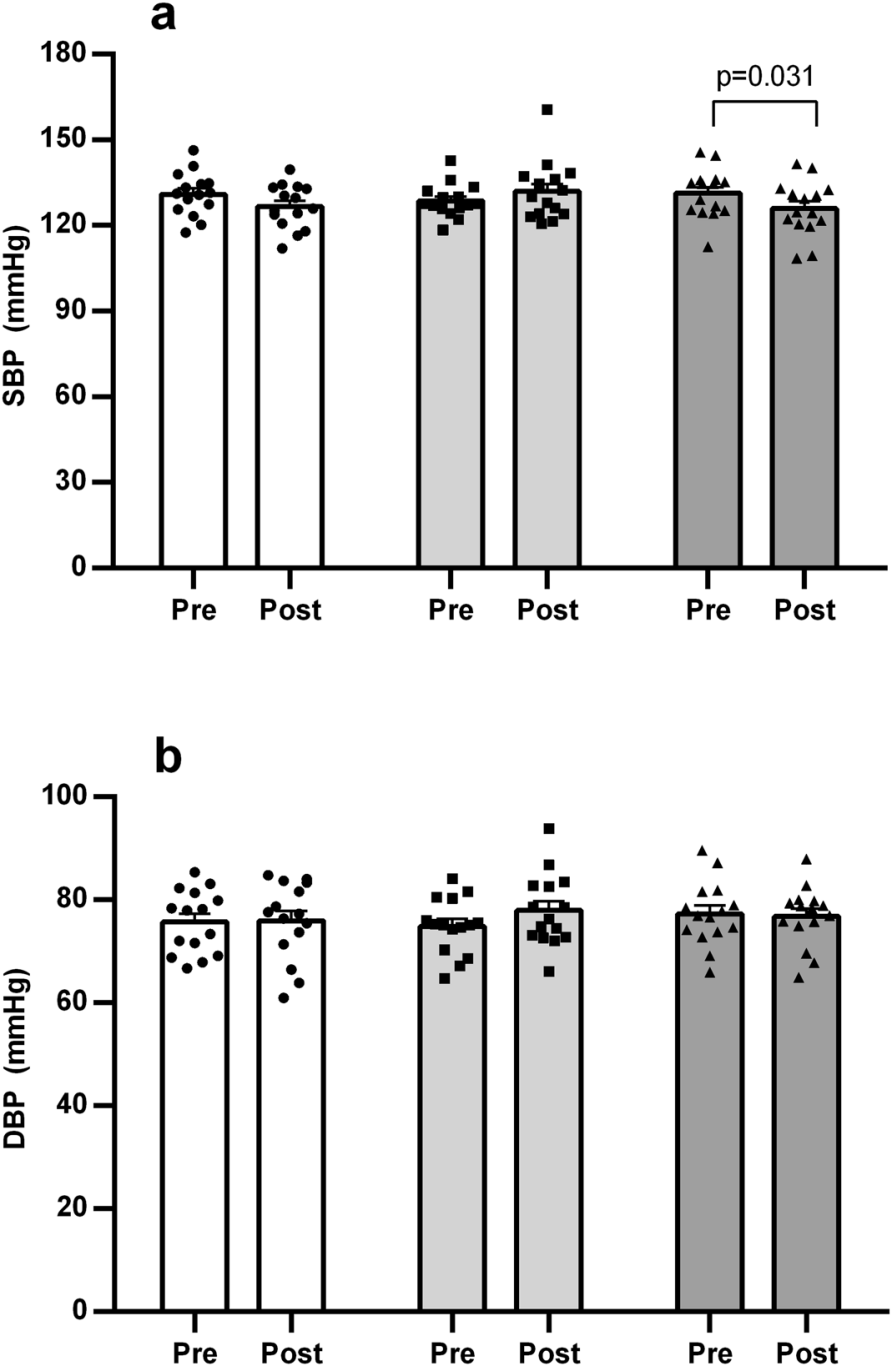
Brachial systolic blood pressure (**a**) and diastolic blood pressure (**b**) before and after aerobic exercise (white bars), resistance exercise (light grey bars), and concurrent exercise (dark grey bars). Data expressed as mean ± SE.

**Figure 3:**
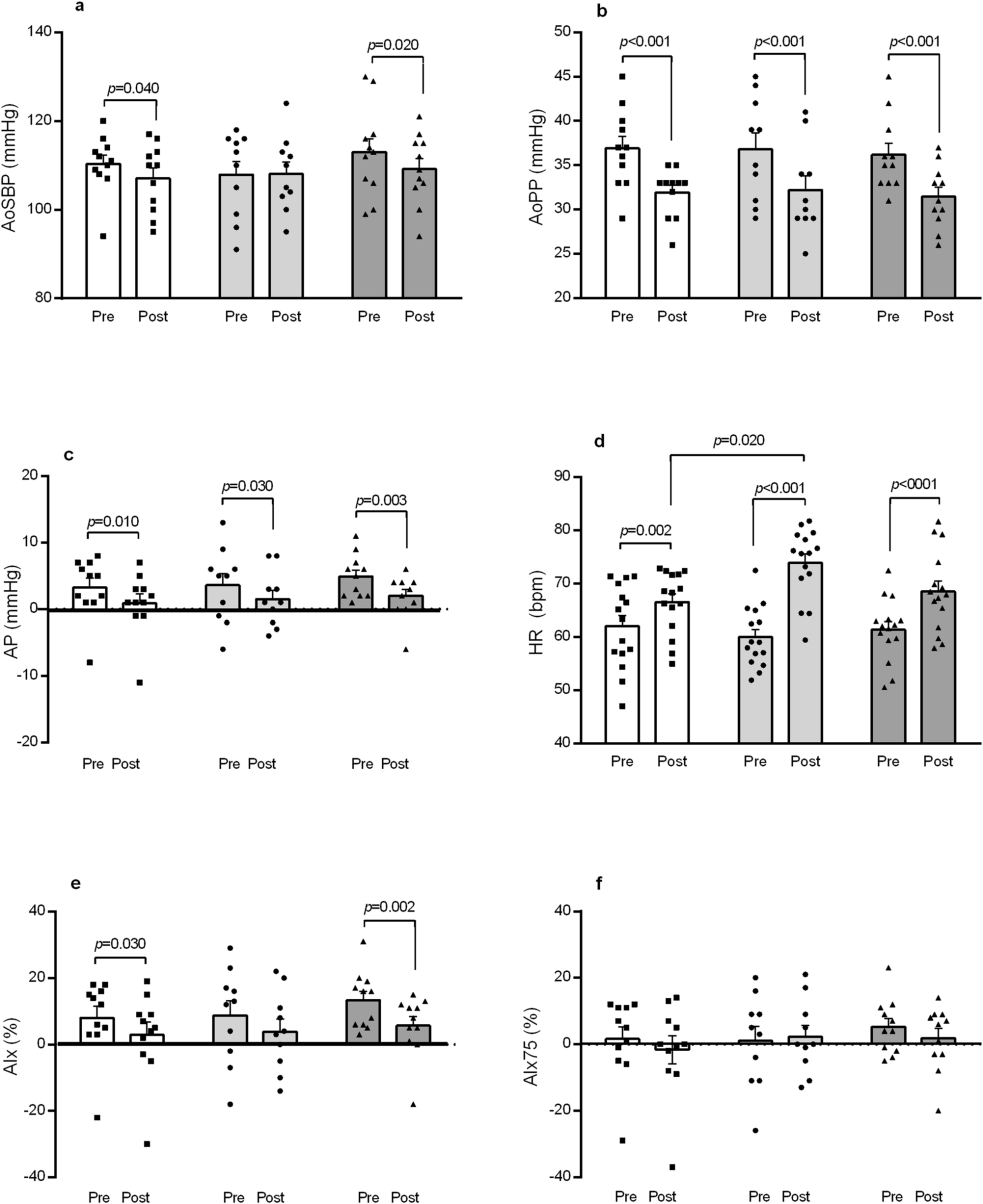
Pulse wave reflection outcomes before and after aerobic exercise (white bars), resistance exercise (light grey bars), and concurrent exercise (dark grey bars). AoSBP (**a**) aortic systolic blood pressure; aoPP (**b**) aortic pulse pressure; AP (**c**) augmented pressure; HR (**d**) heart rate; AIx (**e**) augmentation index; AIx75 (**f**) augmentation index normalized to 75 bpm. Data expressed as mean ± SE.

Figure 2 depicts data for brachial SBP and DBP, while Figure 3 exhibits PWR parameters before and after the exercise bouts. A significant decrease in SBP vs*.* preexercise was detected only after CE (Δ − 5.3 ± 1.9 mmHg). In regards to PWR outcomes, aoSBP lowered pre vs*.* postexercise after AE and CE (1-a), while reductions in aoPP (1-b) and AP (1-c) occurred in the three exercise conditions. Heart rate was always higher vs*.* pre-exercise, being significantly greater in RE than AE (1-d). AIx decreased following AE and CE vs*.* pre-exercise (1-e), while AIx75 did not change after the three exercise conditions (1-f).

Table 2 shows HRV indices before and after exercise. When compared to pre-exercise, iRR decreased vs*.* preexercise after the three exercise conditions, being significantly lower in RE vs*.* AE during all recovery period (*p* < 0.05). The rMSSD equally decreased after AE, RE, and CE (*p* < 0.05). Changes in frequency domain indices occurred only after RE, with increased LF after 25 min postexercise vs*.* rest (*p* = 0.04), and lowered HF at 25 min (*p* = 0.01) and 30 min (*p* = 0.01) vs*.* preexercise. In consequence, LF/HF was higher vs*.* resting conditions from 25 min (*p* = 0.02) to 30 min (*p* = 0.02) after RE.

**Table 2:** Heart rate variability before and after exercise interventions.

	Pre	Post				
		10 min	15 min	20 min	25 min	30 min
**iRR (ms)**						
AE	1002 ± 33.5	810 ± 25.3*	859 ± 22.9*	888 ± 23.0*	908 ± 21.2*	914 ± 21.7*
RE	1025 ± 29.8	693 ± 23.2*^†^	756 ± 21.9*^†^	783 ± 23,2*^†^	813 ± 23.2*^†^	832 ± 23.9^†^*
CE	1011 ± 24.3	776 ± 23.6*	828 ± 25.7*	858 ± 24.1*	879 ± 23.0*	888 ± 20.1*
**rMSSD (ms)**						
AE	53.2 ± 5.1	30.3 ± 3.5*	33.1 ± 4.4*	37.2 ± 4.4*	39.8 ± 3.4*	39.1 ± 3.8*
RE	50.5 ± 5.8	19.8 ± 5.1*	24.9 ± 6.1*	23.7 ± 3.9*	27.9 ± 3.9*	31.4 ± 5.1*
CE	56.2 ± 5.8	23.7 ± 4.0*	29.8 ± 5.6*	33.8 ± 4.6*	37.9 ± 4.2*	40.7 ± 4.2*
**LF (n.u.)**						
AE	55.8 ± 4.6	58.2 ± 4.6	62.2 ± 4.7	58.6 ± 4.5	57.4 ± 4.5	58.5 ± 4.4
RE	60.0 ± 4.3	63.6 ± 4.9	67.0 ± 5.3	66.5 ± 4.0	70.4 ± 3.4*	69.6 ± 4.4
CE	59.0 ± 5.0	66.8 ± 3.9	60.2 ± 4.5	60.4 ± 3.5	64.7 ± 3.1	66.0 ± 4.2
**HF (n.u.)**						
AE	44.2 ± 4.6	41.8 ± 4.6	37.8 ± 4.7	41.4 ± 4.5	42.6 ± 4.5	41.5 ± 4.4
RE	40.0 ± 4.3	36.4 ± 4.9	33.0 ± 5.3	33.4 ± 4.0	29.6 ± 3.4*	30.4 ± 4.4*
CE	41.0 ± 5.0	33.2 ± 3.9	39.8 ± 4.5	39.6 ± 3.5	35.3 ± 3.1	34.0 ± 4.2
**LF/HF**						
AE	1.7 ± 0.3	1.9 ± 0.4	2.4 ± 0.5	2.0 ± 0.4	1.9 ± 0.4	1.9 ± 0.3
RE	2.0 ± 0.3	2.5 ± 0.5	2.9 ± 0.4	2.9 ± 0.6	3.2 ± 0.5*	3.3 ± 0.6*
CE	2.2 ± 0.5	2.9 ± 0.6	2.1 ± 0.4	1.9 ± 0.3	2.1 ± 0.3	2.6 ± 0.4

AE, aerobic exercise; RE, resistance exercise; CE, concurrent exercise; iRR, RR (inter beat) intervals; rMSSD, square root of the sum of successive differences between adjacent normal RR intervals; LF, low-frequency band; HF, high-frequency band; LF/HF, sympathovagal balance; BRS, baroreflex sensitivity.

*Significantly different from pre (*p* < 0.05). ^†^Significantly different vs*.* AE at the same time point (*p* < 0.05). Values expressed as mean ± standard error.

Table 3 presents correlations between changes in central hemodynamic and cardiac autonomic outcomes during recovery from exercise trials. There were positive correlations after CE between AP and AIx vs*.* iRR and parasympathetic modulation reflected by rMSSD and HF, and negative correlations vs*.* LF and sympathovagal balance reflected by LF/HF. As expected, HR had negative correlations vs*.* iRR and rMSSD in AE and CE, respectively, and positive correlation vs*.* sympathovagal balance in AE. Correlations for AIx75 followed the same patterns observed for AIx, but were significant only vs*.* HRV indices in the frequency domain.

**Table 3:** Spearman correlations (*p*-values) between changes in PWR and HRV outcomes (n = 15).

	iRR	rMSSD	LF	HF	LF/HF
**AoSBP**					
AE	0.27 (0.424)	− 0.37 (0.257)	− 0.17 (0.624)	0.17 (0.624)	− 0.19 (0.576)
RE	0.02 (0.973)	0.25 (0.491)	− 0.12 (0.759)	0.12 (0.759)	− 0.13 (0.733)
CE	− 0.04 (0.916)	− 0.20 (0.558)	0.03 (0.939)	− 0.03 (0.939)	0.24 (0.474)
**AoPP**					
AE	0.37 (0.260)	− 0.26 (0.435)	− 0.20 (0.559)	0.20 (0.559)	− 0.08 (0.810)
RE	0.27 (0.448)	0.05 (0.891)	− 0.35 (0.330)	0.35 (0.330)	− 0.12 (0.759)
CE	0.04 (0.916)	− 0.15 (0.667)	− 0.19 (0.570)	0.19 (0.570)	− 0.26 (0.446)
**AP**					
AE	− 0.05 (0.897)	− 0.38 (0.248)	0.05 (0.886)	− 0.05 (0.886)	0.19 (0.579)
RE	0.24 (0.515)	− 0.02 (0.979)	− 0.07 (0.858)	0.07 (0.858)	0.17 (0.659)
CE	***0.62 (0.047)***	***0.67 (0.027)***	***−*** ***0.85 (0.002)***	***0.85 (0.002)***	***−*** ***0.81 (0.004)***
**HR**					
AE	***−*** ***0.69 (0.021)***	− 0.46 (0.152)	0.55 (0.085)	− 0.55 (0.085)	***0.66 (0.030)***
RE	− 0.49 (0.157)	− 0.17 (0.638)	0.07 (0.843)	− 0.07 (0.845)	0.18 (0.624)
CE	− 0.51 (0.111)	***−*** ***0.60 (0.05)***	0.32 (0.337)	− 0.32 (0.337)	0.30 (0.367)
**AIx**					
AE	− 0.18 (0.592)	− 0.32 (0.337)	− 0.07 (0.838)	0.07 (0.838)	0.13 (0.712)
RE	0.24 (0.507)	0.02 (0.965)	0.02 (0.965)	− 0.02 (0.965)	0.24 (0.496)
CE	***0.64 (0.038)***	***0.65 (0.035)***	***−*** ***0.83 (0.002)***	***0.83 (0.002)***	***−*** ***0.76 (0.009)***
**AIx75**					
AE	− 0.43 (0.188)	− 0.52 (0.104)	0.12 (0.728)	− 0.12 (0.728)	0.26 (0.428)
RE	0.08 (0.838)	− 0.07 (0.865)	0.07 (0.865)	− 0.07 (0.865)	0.30 (0.407)
CE	0.46 (0.154)	0.48 (0.141)	***−*** ***0.74 (0.012)***	***0.74 (0.012)***	***−*** ***0.67 (0.029)***

Significant correlations in italic bold.

iRR, RR (inter beat) intervals; rMSSD, square root of the sum of successive differences between adjacent normal RR intervals; LF, low-frequency band; HF, high-frequency band; LF/HF, sympathovagal balance; AoSBP, aortic systolic blood pressure; AoPP, aortic pulse pressure; AP, augmented pressure; HR, heart rate; AIx, augmentation index; AIx75, augmentation index normalized to 75 bpm.

## Discussion

This study compared the after-effects of AE, RE, and CE on indices of PWR and HRV in men with stage-1 hypertension. Additionally, we tested the relationship between changes in PWR and cardiac autonomic modulation reflected by HRV indices. Participants were in general overweight, and exhibited relatively poor aerobic capacity. Glycemic and lipid levels were adequate for people with no health issues. Isolated AE and RE did not provoke acute reductions in BP, but SBP significantly decreased after CE. Overall, PWR outcomes (excepting AIx75) decreased after AE and CE. The impact of acute RE was less evident—significant reductions occurred in AP and AoPP, but not in AoSBP, AIx, or AIx75. Changes in PWR parameters were parallel to decreased parasympathetic modulation after the three exercise conditions, while sympathetic modulation increased following RE. Reductions in AP and AIx after CE correlated with increased sympathetic outflow. Those findings highlight the influence of exercise mode on acute PWR responses reflecting arterial stiffness.

As abovementioned, trials comparing the acute effects of different exercise modes on PWR outcomes are scarce. A recent meta-analysis indicated that exercise mode would be a major determinant of PWR and arterial stiffness changes following acute exercise^4^, with distinct responses resulting from AE and RE. However, none of the 45 reviewed trials compared PWR outcomes following acute exercise bouts of varying modes. Our study contributes with the current knowledge by examining those changes in individuals with elevated BP at rest, and comparing AE and RE performed in isolation and within the same training session (CE).

To our knowledge, there is only a single trial specifically investigating the influence of exercise modes (AE vs*.* RE, but not CE) on arterial stiffness and PWR^12^. Healthy young men performed acute bouts of AE (30-min cycling at 70–75% maximum heart rate) and RE (3 × 10 reps of six upper/lower body exercises at 80–90% of 10-RM). The carotid-femoral pulse wave velocity was essentially stable vs*.* preexercise in both exercise modalities. After AE, AP and AIx also remained quite similar to resting values during 60-min postexercise. On the other hand, AIx75 was greater vs*.* preexercise in early post-intervention (until 10 min), subsequently declining to below resting levels. After RE, AP, AIx, and AIx75 also increased within the first 10-min recovery. Subsequently, AP and AIx gradually declined to resting levels, while AIx75 continued significantly elevated until 40 min post-intervention. Concisely, in this sample of healthy young adults the PWR and arterial stiffness showed a transient increase after RE and remained mostly unaltered after AE. The authors claimed that these findings reinforced the hypothesis of increased PWR and arterial stiffness after RE, while the premise that acute AE would reduce those outcomes was not confirmed. Distinct autonomic adjustments regulating the vasomotor tone might be considered key modulators of these responses.

Our findings in individuals with stage-1 hypertension did not concur with those results. AP and AoPP significantly decreased after acute AE, RE, and CE, meaning that aortic central pressure and cardiac afterload lowered regardless of the exercise modality. Moreover, AoSBP and AIx decreased after AE and CE, which is suggestive of reduced systemic arterial stiffness. Individuals with higher BP usually exhibit greater levels of arterial stiffness^1^. In people with elastic arteries, the reflected wave travels backward slowly (against the blood flow direction) and arrives at the aortic root during late systole or early diastole, which favors the filling of coronary vessels^37^. On the other hand, the reflected wave travels faster in individuals with stiffer arteries, arriving at the aortic root in the next systole. In this case, AP summates with the forward wave, increasing AoPP due to a greater difference between AoSBP and aortic DBP^37^. Hence, it is feasible to think that the BP level at rest might be a determinant of PWR responses to acute exercise.

The present results give support to the hypothesis that, at least in individuals with elevated BP, postexercise reductions in PWR might occur after AE and CE. Reductions were not detected after RE, but it should be noted that there were no increases either, as previously reported in normotensive individuals^12,22,26,44^. While reductions in AIx after acute AE have been previously demonstrated^10,17,45^, data regarding RE are few and inconsistent^12,26,46^. A recent meta-analysis^4^ including trials mostly developed with healthy individuals reported significant reductions in AIx following acute AE but not RE, while increases in AIx75 were detected after both AE and RE. On the other hand, some trials found reductions in arterial stiffness after acute RE, at least in protocols applying exercises with low- to moderate intensity for the lower body^22,47^, or in samples with elevated PWR at rest^15^. Our RE protocol concurred with these last conditions (i.e., moderate load in the leg extension exercise and individuals with elevated BP), which may help to explain the decrease in aortic wave reflection represented by AP. However, this reduction seemed to be proportional to changes in AoPP, and therefore were not suggestive of reduced arterial stiffness. Accordingly, significant reduction in AIx was not observed after RE. Decreases in AoPP and AP possibly resulted from increased DBP, since there was no reduction in AoSBP. This finding reinforces the idea that RE has little impact on PWR in comparison with AE or CE. Previous studies suggested that the dominant vasodilator effect of AE would be the primary mechanism responsible for alterations in the reflected wave after both AE and CE^10,18,44^.

Indices of wave reflection following exercise are influenced by multiple factors, including heart rate, left ventricular ejection duration, and arteriolar vasomotor tone resulting from sympathetic outflow^4,22,24^. Greater sympathetic outflow increases peripheral vasoconstriction, which contributes to enhance the reflected wave intensity^24^. In the present study, a significant depression of parasympathetic modulation reflected by rMSSD persisted during the 30-min recovery, regardless of the exercise modality. After RE, this was concomitant to a slight increase in sympathovagal balance reflected by LF:HF. Although a vagal withdrawal has been detected after AE and CE, an increased sympathetic modulation was not observed, which might have tempered the vasoconstriction drive in these modalities vs*.* RE^25,27^. The stronger exercise pressor reflex during RE attributable to the compressive effects on blood vessels^48^, may help to explain its greater impact on sympathovagal balance vs*.* AE and CE.

Moreover, shear stress patterns affecting vasoconstriction seem to depend on exercise mode^25,49^. Most trials reporting a reduction in AIx following AE linked this finding to increased peripheral vasodilation, rather than to a reduced sympathetic vasoconstriction^9,10,12,45^. The few studies that observed reductions in PWR after acute RE, also suggested that this would be secondary to changes in the transit time of the reflected wave^15^ due to peripheral vasodilation mediated by local metabolite accumulation^10,47^. Vasodilation probably occurs when endothelium-mediated effects override the sympathetic vasoconstrictor activation^25^. In short, our findings reinforce the premise that attenuations in PWR may result from greater vasodilation after AE (performed in isolation or within CE) vs*.* RE^10,18,44^. Since the magnitude of wave reflection is influenced by a mismatch between central and peripheral vasomotor tone^24^, the greater peripheral vessel constriction after RE may have resulted in increased wave reflection vs*.* AE or CE.

This rationale also helps to explain the negative correlations found between changes in PWR vs*.* autonomic modulation. AP and AIx after CE were inversely associated with sympathetic indices and directly correlated with indices with parasympathetic predominance. This means that favorable responses in PWR occurred despite of an increased sympathetic vasoconstrictor drive, which rejects our hypothesis that changes in autonomic control would be potential mechanisms of acute PWR responses to exercise. Actually, prior research has reported lowered vagal and increased sympathetic activity parallel to acute reductions in BP after different exercise modes^28,50^. This response probably occurs to offset the BP lowering and to compensate for a resetting in baroreflex^25,27^. In short, reductions in BP following acute exercise seem to depend on peripheral factors reflecting the ability of vasodilation to counteract the autonomic reaction to bring BP back to pre-exercise levels.

Our data indicated that the vagal reentry was delayed after all exercise modalities and sympathetic modulation during recovery increased in RE. The BP acutely decreased only after CE, which was the modality showing the stronger correlations between PWR and HRV indices. There is evidence that changes in autonomic control as a mechanism of postexercise hypotension are mediated by the amount of muscle work via exercise pressor reflex^25,27,50^. On the other hand, the shear stress leading to local vasodilation is also related to the exercise volume^10,51^. Therefore, it is not surprisingly that SBP decreased only after CE, which applied greater exercise volume vs*.* AE and RE. The BP reduction might have triggered a compensatory autonomic reaction to restore the resting levels and counteract the peripheral vasodilation, thus lowering peripheral resistance and arterial stiffness. It is feasible to speculate that the sympathetic response would be proportional to the level of local vasodilation, and this helps to explain why correlations between PWR and autonomic indices were stronger in CE vs*.* AE and RE. These results are suggestive that autonomic changes after acute exercise would be rather a consequence than cause of reductions in PWR outcomes.

Finally, it is worthy to notice that recovery of vagal modulation was similar after AE and CE, which disagrees with a previous trial^28^ reporting longer parasympathetic withdrawal after CE than AE. However, in that study, the resistance component was performed later in the CE session, while AE was presently performed after RE. Differences in data from the two experiments suggest that the order of exercise modes within CE may influence the autonomic recovery, perhaps due to the release of catecholamines and metabolite accumulation typically observed during RE^15,42,47^. Further research is warranted to investigate the potential influence of exercise order within CE upon acute PWR and autonomic responses.

This study has limitations. Firstly, the sample included only overweight men with elevated BP, which limits the generalization of our findings to other populations. Another major limitation results from differences between the exercise protocols, which were not matched for the time or overall volume. While AE was in line with recommendations for health-related exercise prescription, RE was not. As abovementioned, our facilities were restricted and we were not able to meet the current recommendations of multiple exercises for the upper and lower body. Additionally, CE included both the entire workouts for AE and RE, meaning significantly more volume. This option is justified by the limited amount of RE and the desire to reproduce what is actually done in training centers, thereby marking the differences between exercise modalities. The fact that PWR responses were quite similar across conditions suggests that this option was appropriate. If the interventions had been matched by volume, there would remain doubt about the relative role of this variable vs. exercise modality. Moreover, we have not directly measured changes in arterial pulse waves; central pressure indices have been indirectly derived using a transfer function. However, applanation tonometry has been widely used in studies investigating arterial stiffness in different populations and research sets, being considered the gold standard in pulse wave analysis^4,36,52,53^. Differences in exercise intensity may influence HRV^39^ and bias due to this feature must be considered in future studies. It must be also remarked that reductions in AIx must be interpreted with caution, since they may just reflect increases in HR. Although the use of AIx75 as a correcting factor in acute studies has been argued^38^, we must acknowledge that this variable remained unaltered after all exercise modalities, irrespective of changes in Aix. Further studies with larger and distinct samples are therefore necessary to confirm our findings and extend their clinical applications.

## Conclusion

Our data suggested that AE, RE, and CE were capable to produce acute reduction in central aortic pressure pulse, while only AE and CE reduced PWR indices in overweight men with stage-1 hypertension. These effects were concomitant with lower parasympathetic modulation and, at least after RE, increased sympathovagal balance. Reductions in PWR correlated with increased sympathetic and lowered vagal modulation in CE. This is suggestive that autonomic fluctuations had little influence on changes in PWR after acute exercise, and that local vasodilation factors may have a predominant role. Further investigations are warranted to ratify these findings by concomitantly assessing central and peripheral factors related to arterial stiffness. Additionally, research is needed to determine the relative role of PWR responses to acute exercise of different modes in provoking adaptations to chronic exercise training.

## Data Availability

Data is available on request and interested researchers may address data access requests to the contact mariofneves@gmail.com.

## Abbreviations

PWR: Pulse wave reflection
AE: Aerobic exercise
RE: Resistance exercise
CE: Concurrent exercise
BMI: Body mass index
CPET: Cardiopulmonary exercise testing
HDL: High-density lipoprotein
LDL: Low-density lipoprotein
GFR: Glomerular filtration rate
VO_2_: Oxygen uptake
GET: Gas exchange threshold
RM: Repetition maximum
aoSBP: Aortic systolic blood pressure
aoPP: Aortic pulse pressure
AP: Augmented pressure
AIx: Augmentation index
AIx75: Augmentation index normalized to 75 bpm
HRV: Heart rate variability
iRR: R-R intervals
rMSSD: Square root of the sum of successive differences between adjacent normal R–R intervals squared
LF: Low-frequency power
HF: High-frequency power
